# Innovative gamification and outreach tools to raise awareness about antimicrobial resistance

**DOI:** 10.3389/fmicb.2022.977319

**Published:** 2022-09-15

**Authors:** Antonio Tarín-Pelló, Elisa Marco-Crespo, Beatriz Suay-García, Carolina Galiana-Roselló, José I. Bueso-Bordils, María-Teresa Pérez-Gracia

**Affiliations:** ^1^Departamento de Farmacia, Universidad Cardenal Herrera-CEU, CEU Universities, Valencia, Spain; ^2^Departamento de Comunicación e Información Periodística, Universidad Cardenal Herrera-CEU, CEU Universities, Valencia, Spain; ^3^Departamento de Matemáticas, Física y Ciencias Tecnológicas, Universidad Cardenal Herrera-CEU, CEU Universities, Valencia, Spain

**Keywords:** antimicrobial resistance, scientific awareness, health communication, antibiotics, education, AMR awareness, citizen science, antimicrobial stewardship

## Abstract

Since 2017, the SWICEU team has developed various informative actions and innovative gamification supports to educate and raise awareness about antimicrobial resistance (AMR) and the correct use of antibiotics among the general population especially among young people. This case study presents the results obtained in the last 5 years with the strategies carried out by this team, composed of students and professors of Health Sciences, Industrial Design Engineering, and Communication Sciences at CEU Cardenal Herrera University (CEU UCH) in Valencia (Spain). Over the past 5 years, playful educational supports have been developed to make the health problem of bacterial resistance and the action of antibiotics more understandable among young people. The dissemination media used, with the same objective of teaching and raising awareness about AMR in a creative and innovative way, have been selected according to the trends in digital communication and use of scientific and health content provided by the most recent studies carried out among the Spanish population. These strategies have included decalogues or “tips” with useful advice, infographics, YouTube videos, Twitter threads, online challenges on Kahoot, stories on Instagram, use of QR codes, etc. These actions have also obtained diffusion in the media and have been awarded by different national and international entities. The good results obtained in the case under study allow us to establish recommendations for the design of innovative educational gamification and dissemination supports on AMR, especially aimed at younger audiences.

## Introduction

Antimicrobial resistance (AMR) has become a serious global health problem (European Centre for Disease Prevention and Control (ECDC), 2020; Tarín-Pelló et al., 2022a). The World Health Organization (WHO) has listed AMR as one of the top 10 public health threats facing humanity, potentially reaching 10 million deaths annually by 2050 (World Health Organization (WHO), 2019). The most current data can be found in the analysis by Murray et al. where they explain that in 2019 the global burden associated with drug-resistant infections evaluated in 88 pathogen-antimicrobial combinations was approximately 4.95 million deaths, of which 1.27 million were directly attributable to resistance to these compounds. That is, if drug-resistant infections had been sensitive to treatments, 1.27 million lives would have been saved (Murray et al., 2022).

The World Health Assembly approved in May 2015 the global action plan on antimicrobial resistance (World Health Organization (WHO), 2016). Within this plan are strategic objectives focused on improving awareness and knowledge of antimicrobial resistance, as well as optimizing the use of these drugs (Pharmaceutical Research and Manufacturers of America (PhRMA), 2021). Both socially and professionally, continuous training is necessary through different methods, such as the use of media, conferences by specialists in the field, and Antimicrobial Optimization Programs (PROA) (Dyar et al., 2016). The importance of communicating, informing, and disseminating the seriousness of this threat to the population is part of the challenge that we must address, since it is through these approaches that it becomes possible to ensure the prevention and treatment of infectious diseases by means of effective and safe drugs (Spellberg et al., 2013; World Health Organization (WHO), 2016; Collignon et al., 2018). Therefore, it is necessary to design novel and original strategies to educate and raise awareness in society on AMR and the rational use of antimicrobials.

An example of education and awareness of this problem is the MicroMundo project, which is also focused on attracting young students to enter STEM (Science, Technology, Engineering, and Mathematics) careers (Global Leaders Group on Antimicrobial Resistance, 2021). Sponsored by the Spanish Society of Microbiology (SEM), more than 30 universities in Spain and Portugal are currently involved, including CEU Cardenal Herrera University under the name SWICEU (Bueso-Bordils et al., 2020).

The SWICEU project has been characterized since its inception, 5 years ago (2017), by having as its main goal to provide creative and innovative responses to raise awareness of the global health challenge posed by AMR, a priority challenge for the WHO, through a Service-Learning (LS) strategy. It has also stood out for awakening scientific vocations among pre-university and university students, the “Fleming of tomorrow” program has also stood out for awakening scientific vocations among pre-university and university students, through experience and gamification, by participating in a real scientific experiment to search for new antibiotics in the natural environment. From the university, scientific events have been organized and various recreational supports have been created to promote curiosity about this health challenge, also while playing and in a fun way. Another of the defined objectives has been to raise awareness in society with dissemination campaigns in different languages to understand the role we all play in making good use of antibiotics so that they preserve their effectiveness in the future. Finally, it is important to detail the objective of strengthening the links between future health, design, and communication professionals, in a transversal and interdisciplinary experience, to make visible the importance of collaboration between these fields in issues related to public health. In this paper, we will show the scope of the SWICEU project, demonstrating that it is possible to raise awareness in society in a pedagogical, didactic, and playful way about these challenges that not only concern health professionals, but the entire population.

## Materials and methods

The SWICEU team has been the first of the international networks Small World Initiative (SWI) and Tiny Earth (composed of more than 200 universities from 12 countries) to incorporate Communication students to the team formed by university students of Health Sciences, for the dissemination of the global health challenge posed by AMR. It has also counted on Design Engineering students for the development of attractive graphic formats for the dissemination and gamification supports created. The combination and diversity of students from different university degrees, has provided the SWICEU team with multiple approaches to make the AMR problem and the rational use of antimicrobials, two topics of interest to the general population, achieving a record adapted to all audiences unfamiliar with medicine and infectious diseases.

### Dissemination strategies

During the 5 years of development of the SWICEU project, the team has designed several communication and awareness campaigns for the dissemination of the health problem posed by AMR. For the selection of the most appropriate dissemination supports, the SWICEU team has analyzed the media and social media consumption trends detected in the most recent studies among young people and the general population in Spain, especially in relation to health content.

According to the latest Survey of Social Perception of Science and Technology (Fundación Española para la Ciencia y la Tecnología (FECYT), 2021), young Spaniards from 15 to 24 are the age group that claims to have a greater interest in scientific content (21.9%) and that participates more in outreach activities (20.7% in the last year). Medicine and Health are the scientific topics that arouse the most interest (3.79 out of 5). In this age group, the Internet is the most used source of scientific information (79.6%), ahead of television (78.1%), unlike the average of the population.

On the Internet, the main sources for information about science among “generation Z,” to which this age group belongs, are social media (85.9%), YouTube videos or similar sites (69.2%), and digital mass media (56.8%), which present the lowest percentage of use in this age group. In the case of health issues, 80.3% of young people in this age group claim that they get their information from the Internet and only 42.7% from a doctor or nurse.

In order to select the appropriate Internet channels for information on topics such as health, the most recent studies on the use of the Internet, such as the 24th Navigators on the Internet (Asociación para la Investigación de Medios de Comunicación (AIMC), 2022), should be taken into account. This study points out, for example, that online video viewing (YouTube, Twitch, or TikTok type) is the second most performed audiovisual activity by Spaniards (63.1%), only 1.1% behind watching movies and fiction series on platforms such as Netflix (64.2%). According to this same AIMC study, conducted in March 2022, Instagram and Twitter are the second and third most used social networks by Spaniards, behind Facebook, which continues to fall in use. It also reveals that QR code scanning has grown from 35.2% in 2013, to 77.7% in 2021. Accessing extended information on a topic is the second most used activity through QRs, not only used to view a restaurant menu or to display the Covid-19 passport. In general, among the 10 things we do most on the Internet, reading current news is the second (75.5%), and searching for health information is the seventh (40.8%).

For its part, the Social Networks Study 2021 (IAB Spain and Elogia, 2021) indicates that the most used social networks in Spain by “generation Z,” which includes young people aged 16 to 24, are WhatsApp and Instagram (86%), YouTube (79%), and Twitter (63%). This study also reflects that, although we use social media mainly for entertainment (81%) and to interact with others (72%), there is a third use not far behind percentagewise: to be informed (66%). Hence the need to use these digital platforms to offer truthful and contrasted information, especially to younger people and especially when what they are looking for is health information. These data make it possible to design more effective strategies by selecting the mass media and social media most used by the targets of health awareness actions, such as AMR.

**Figure 1:**
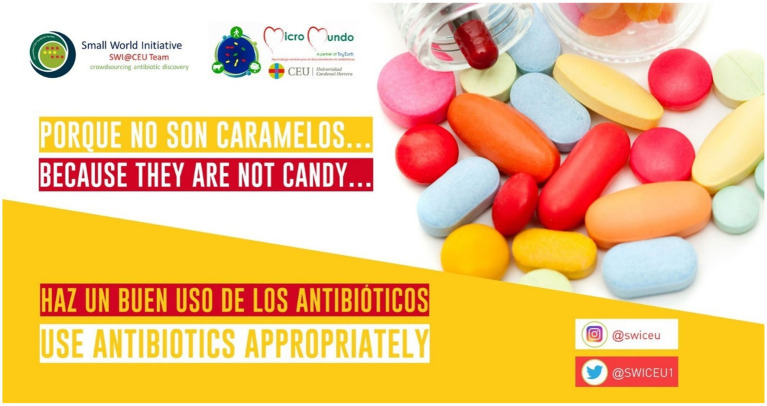
2019 campaign poster “Because they are not candy.. use antibiotics appropriately.”

**Figure 2:**
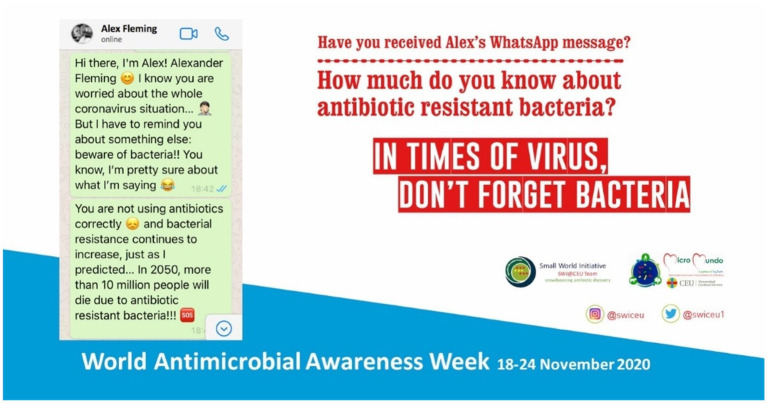
2020 campaign poster “In times of virus, do not forget bacteria.”

The SWICEU team has based itself on the latest editions of the aforementioned studies (IAB Spain and Elogia, 2021; Fundación Española para la Ciencia y la Tecnología (FECYT), 2021; Asociación para la Investigación de Medios de Comunicación (AIMC), 2022) to design an annual awareness campaign on AMR and prudent use of antibiotics, on the occasion of the European Antibiotic Awareness Day, which is celebrated every November 18, and the WHO World Antimicrobial Awareness Week (WAAW), which is celebrated from that day onwards. These communication campaigns were designed for the general population, but especially aimed at university and pre-university students, have been:

-  2018: Campaign “Diez consejos para evitar la resistencia bacteriana a los antibióticos”/“Ten tips to avoid antibiotic resistance” (Actualidad CEU, 2018). Available at: https://youtu.be/HJx3cKIA1yM
-  2019: Campaign "Porque no son caramelos… haz un buen uso de los antibióticos"/"Because they are not candy… use antibiotics appropiately" (Actualidad CEU, 2019; Figure 1).
-  2020: campaign "En tiempos de virus, no olvides las bacterias"/"In times of virus, don't forget bacteria" (Actualidad CEU, 2020; Figure 2).
-  2021: Campaign "Esto NO es un juego, ¿juegas?"/"This is NOT a game, do you play?" (Actualidad CEU, 2021a). Available at: https://youtu.be/LfL09wPP7D0

In response to the aforementioned trends in the use and consumption of content on the Internet and social media, these campaigns have used various communication channels and formats to raise awareness of AMR. The 2018 campaign was based on the use of YouTube videos with a decalogue of advice or health tips for the use of antibiotics, in English and Spanish. These videos were shared on Instagram and Twitter. In 2019, these tips were edited into infographics, and translated into more than 20 languages, with which Twitter threads were developed. New videos were edited and shared on YouTube, Twitter, and Instagram, in stories format.

In 2020, the campaign followed the graphic design of the WhatsApp environment, using the formats and emoticons typical of this social network. A social experiment was shared in video format for YouTube, posing a “time travel” to the year 2050, when it is estimated that AMR deaths will reach 10 million per year. In addition, online quizzes were posed, through the Kahoot platform, with questions about knowledge of AMR and antibiotic use.

In 2021, a video teaser was produced for YouTube inspired by the aesthetics and plot of one of the most watched series on Netflix (“The Squid Game”) to invite participation in various online challenges, this time through Forms. Also in these campaigns, all the content generated was shared on Twitter and Instagram.

### Educational gamification supports

Multiple recreational supports have been created with innovative and attractive formats for the younger generations such as card games, board games with characters and roles related to Microbiology, a portable escape room or “escape box,” as well as participation in real scientific experiences and events. The integration in the SWICEU team of Communication and Design Engineering students has allowed for the development of very attractive contents for the most current dissemination and gamification supports.

#### Guide “create your microstory”

This is a guide with fact sheets on the scientific characteristics of various bacteria and antibiotics, in a format in which each microorganism and drug is presented as a character for a fictional narrative in any format. In this way, it is possible to approach science and the problems of AMR through an entertaining and simple reading. However, the most interesting component of this guide is the ability to create “micro-stories” with bacteria and antimicrobials as “characters,” providing the reader with suggestions for the creation of different narrative formats such as comics, video games, songs, videos, short films, series, short stories, musicals, etc.

**Figure 3:**
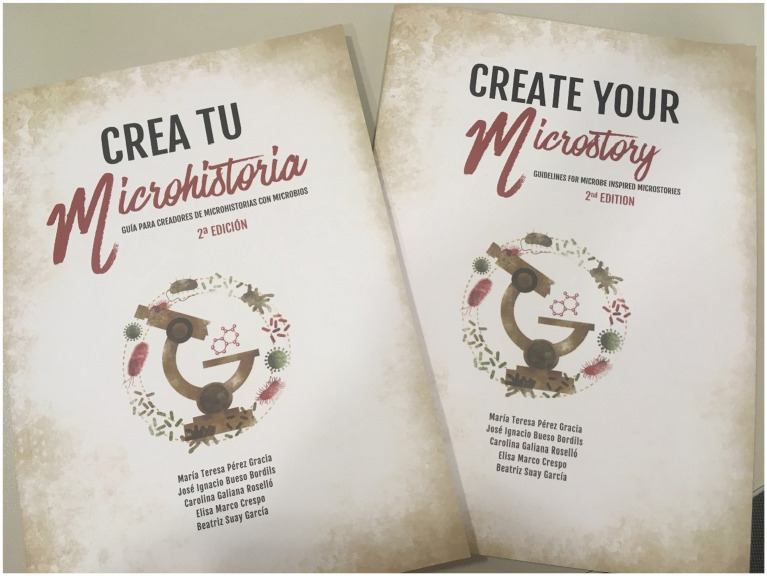
Second edition of “Create your microstory. Guidelines for microbe inspired microstories.”

With two editions published, both in Spanish and English (Figure 3), this initiative is framed to raise awareness of the priority bacteria that the WHO has indicated have demonstrated higher levels of resistance to various generations of antibiotics and that pose a public health threat. All this with an attractive and entertaining design, suitable for all audiences.

#### Bookmark in 20 languages

**Figure 4:**
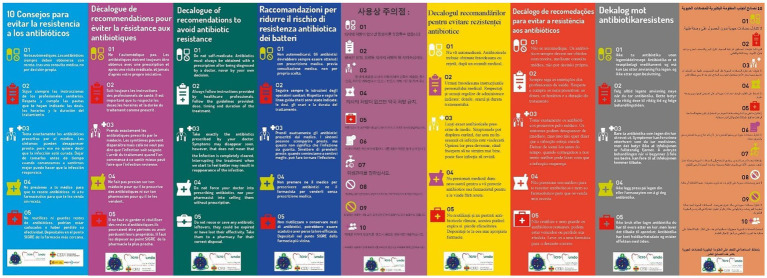
Infographics of the decalogue of recommendations to avoid antibiotic resistance in several languages. Bookmark format.

With a decalogue of recommendations to avoid antibiotic resistance developed by the SWICEU team, and following the recommendations of public health agencies, these bookmarks with infographics for the “Create your microstory” guide have been translated into 20 different languages (Spanish, French, Italian, Russian, Romanian, Norwegian, Portuguese, Korean, Vietnamese, Arabic, etc.; Figure 4). This simple format allows us to understand that we can all contribute to a rational use of antibiotics, as well as to take a few easy steps in our daily lives to avoid and prevent infections.

#### Deck of cards “2050 ¡infection!” two games in one

**Figure 5:**
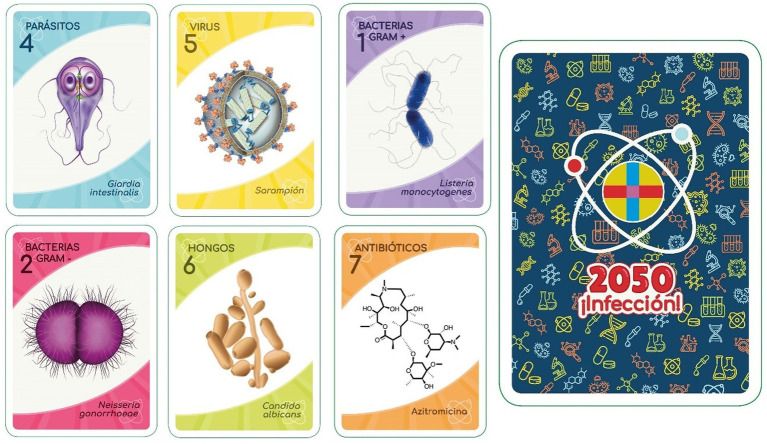
Cards belonging to the “2050 Infection!” deck.

The SWICEU team has also designed a 56-card deck with characters and roles related to microbiology and antibiotic resistance (bacteria, antibiotics, plasmids, superbugs, microbiota, scientist, etc.; Figure 5).

The deck has two game options: one is a role-playing game, where there are two main teams, the bacteria and the antibiotics, which must eliminate the opposing team to win (symbolizing the success of the bacteria in acquiring resistance to existing antibiotics, or the achievement of the antibiotics in adequately treating infections). In addition to these two teams, it is possible to play with up to 11 characters (for example, Superbacteria, Plasmid, Journalist, and Symbiont.) that perform different functions related to reality (for example, the “Plasmid” card provides resistance to a bacterium, preventing it from being eliminated by antibiotics).

The second option is the clan game, made up of 42 cards from the deck and whose objective is to obtain the highest number of clans in a game. The deck presents a total of six figures belonging to each of the seven clans (Gram-positive bacteria, Gram-negative bacteria, and bacteria of the *Enterobacteriaceae* family, antibiotics, viruses, parasites, and fungi). To win the game, the player must request a card from one of the clans in his hand. If successful, the player may draw the corresponding card from his opponent. If the opponent does not have a card of the requested clan in his hand, the player must pass his turn to the next player. Once all cards of a clan have been obtained, they are displayed on the table to show that the player has obtained a clan.

Both styles of game allow the acquisition of knowledge on the rational use of antibiotics and the problems of AMR, as well as bringing Microbiology, science, and research closer to all audiences in a fun and attractive format, through the names, images, and functions found in each card.

#### “Superbugs” deck of cards in which participants become researchers who must gather the necessary laboratory elements to find a new antibiotic

**Figure 6:**
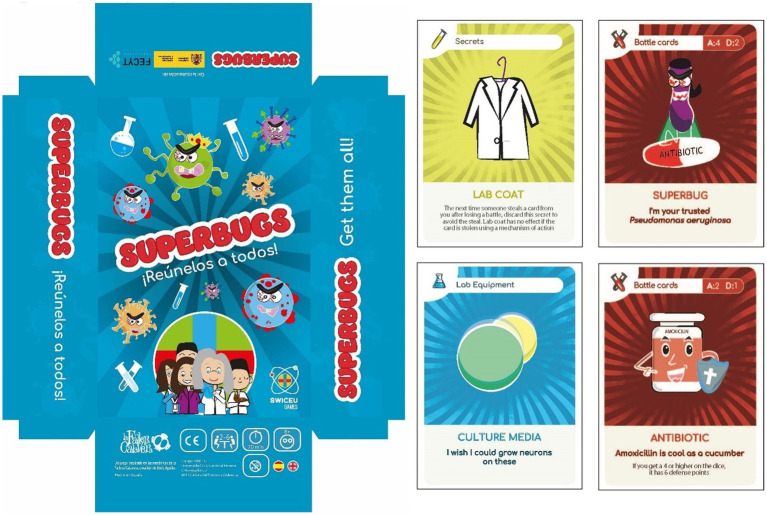
Box and card design of the “Superbugs” card game.

In this card game created by the SWICEU team, players assume the role of scientists dedicated to the discovery of new antibiotics. To do so, the player will have to obtain the necessary laboratory material to create a new molecule with antibacterial activity before the other players do. Made up of 170 cards, this game has been designed with Microbiology characters and concepts, focusing on antibiotic resistance. The deck is divided into three types of cards: bench cards (for example, microscope, culture media, and micropipettes), combat cards (for example, bacteria, superbacteria, and antibiotics), and mechanisms of action cards (for example, antibiogram, cryotubes, FFP2 mask). The game is won when a player gets 5 bench cards that allow him to develop a new antimicrobial before the rest of the scientists/players. To be the first to achieve it, the player must use the combat cards and the mechanism of action cards, which have a description for a proper understanding (Figure 6). This game allows the acquisition of knowledge on the rational use of antibiotics, the importance of finding new antimicrobials, and the problem of AMR, as well as bringing microbiology, science, and research closer to all audiences, especially the youngest, in a fun and attractive format.

#### “Cluedo” style game

This cluedo devised by the SWICEU team takes place in a laboratory setting with characters and situations related to the field of microbiology (scientists working with bacteria, antibiotics, viruses, fungi, parasites, multidrug-resistant bacteria, etc.). The rivalry between researchers to be the first to find a new antibiotic to save humanity generates different possibilities of mysterious murders, possible culprits and probable causes of death that must be investigated in each game, putting into practice the knowledge of Microbiology that the game itself provides.

#### Portable escape room or “escape box”

**Figure 7:**
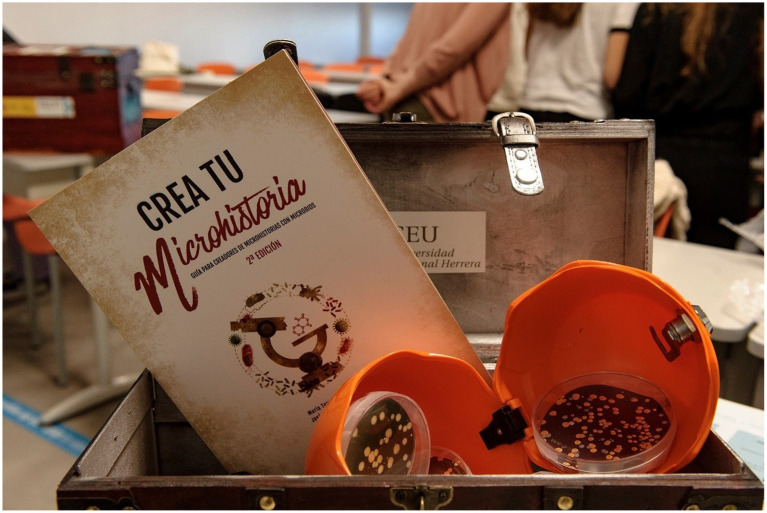
Box and objects for testing the escape box on AMR, designed by the SWICEU team.

In this game, players are presented with an unusual situation. They are in the year 2050, humanity is on the verge of extinction due to the reckless use of antibiotics and the participants must help humanity to survive by opening a mysterious chest found in a laboratory located in Japan for the research of multi-resistant bacteria. To do so, they will have to solve the tests and riddles found inside some trunks (Figure 7). This chest contains all the elements of the game, from safes and keys to test tubes with reagents and ultraviolet light. The objects in the mystery chest are opened and deciphered with various keys, obtained from videos and websites, which participants must consult with their cell phones to find or enter passwords. Some of these keys are also hidden in the decalogue of advice on the proper use of antibiotics and in the guide ‘Create your microhistory’ prepared by the SWICEU team (Figures 3, 4). All of this is related to Microbiology, more specifically to antibiotic resistance and the practices carried out during the SWICEU project. Thanks to its portable design, it can be moved and prepared anywhere. This game has been introduced during the 2021–22 academic year in seven educational centers participating in the SWICEU project with a great reception by the 104 participating 16–17 years old students (Actualidad CEU, 2021b; Tarín-Pelló et al., 2022b).

## Results

The dissemination and gamification activities of the SWICEU team in these 5 years described so far have achieved satisfactory results in several dimensions. In terms of dissemination in the media, they have been published in more than 40 different Spanish media, both general media (ABC, La Vanguardia, Levante, Heraldo de Aragón, 20 Minutos, COPE, Onda Cero, etc.), as well as specialized in health (Correo Farmacéutico, Con Salud, BioTech Spain, etc.) and also in scientific dissemination sites (DICYT, RUVID, Madri+d, NCYT Amazings, etc.), with the publication of press releases, interviews on radio programs and television reports. In the 2020–21 academic year alone, the total audience of the media that disseminated news about the activities of the SWICEU team amounted to 3,362,938 people, with an estimated economic value of the dissemination, according to the advertising rates of each media outlet, that would exceed 53,043.28 euros per year, an indicative average figure that can be extended to the remaining 4 years of the project’s development. In addition, the informative contents on the activity of the SWICEU team between 2017 and 2022 have reached a total of 185,475 visits in the scientific news section of the CEU UCH website *Actualidad CEU*, where they are the most viewed contents in this site.

Regarding the dissemination on social media, the impressions on Twitter of the contents shared on this social network, through the profiles @SWICEU1 and @CienciaUCHCEU, are well over half a million, with a total of 619,220 impressions of the tweets and threads shared. On Instagram, there has been a 202% increase in followers between the first and second year of its creation. The main activity of the profile in this social network is concentrated in the months of February and March of each course, coinciding with the face-to-face experiments in the participating schools and institutes. With an average of 10 posts and 15 stories during these months, an average of 8,700 monthly impressions and more than 400 interactions are achieved. The majority age group that interacts with this profile is 18 to 24 years old (45.8%). And on YouTube alone, the videos shared have reached 8,843 views, between those made by the SWICEU team and the “micro-stories” created in video by students from schools and institutes participating in the project. In addition to the views on the CEU UCH YouTube channel itself, we should add the views of these contents on the scientific news site of the CEU UCH website *Actualidad CEU*, as well as on social media, where the videos have also been shared.

Another notable result of the dissemination actions are the awards won by the SWICEU team:

-  Prize in the 3rd Annual Do Something About Antibiotics Challenge, from the Small World Initiative (SWI) network, awarded by Yale University and the Spanish Society of Microbiology (Small World Initiative, 2018), “for their extraordinary contribution to the objectives and values of the project with the production of excellent divulgative videos made by their students” during the World Antibiotic Awareness Week (12–18 November 2018).
-  Winners of the Antibiotic Awareness Campaign 2018 Overall Winner, from the Tiny Earth Network, awarded by the University of Wisconsin-Madison’s, home of this network composed of more than 10,000 students from 15 countries (Tiny Earth, 2018).
-  Three awards in Tiny Earth Network’s 2019 Antibiotic Awareness Campaign (Tiny Earth, 2019):

o  1st prize in the “Public Service Messages” category for Twitter infographics on bacterial resistance to antibiotics.
o  3rd prize in the “Video” category for a short film emulating the silent cinema format to stage 10 situations in which these drugs are misused, offering the appropriate recommendation (Universidad CEU Cardenal Herrera, 2019).
o  3rd prize in the “Miscellaneous” category, for the stand installed on the University campus to inform about the proper use of antibiotics under the slogan “Porque no son caramelos/Because they are not candy” (Actualidad CEU, 2019).

The gamification educational supports described in this article have won awards for teaching innovation and external funding. The awards obtained have been the Innova(c)tion in Teaching and Services Award for the 2017–18 academic year at CEU Cardenal Herrera University, in the category Innovac(c)ion in the Classroom. And the Award for the best teaching innovation project at the II Congress of Educational and Teaching Innovation CEU-CIED, among 194 projects submitted by 644 teachers from the educational centers of the CEU group in Spain.

The games developed by the SWICEU team have brought to the project an innovative approach that has been very well received by the university and pre-university students who have participated in the Tiny Earth project. Over the past year, 470 students from second-grade schools have received some of the card games, in addition to participating in the “escape box” demonstrating enthusiasm for these new forms of recreational training.

Since its implementation, the SWICEU project has also received funding from the CEU UCH’s CEU Innova program for Innovation and Improvement of Teaching Quality, in the modality of Innovation Projects based on LS. This funding has been obtained in five consecutive calls.

## Discussion

The impact of the project relies on the formative effects of a participatory teaching experience, integrated in an international project, with an important social projection (Bueso-Bordils et al., 2020). From the academic point of view, university students have learned the importance of working in a multidisciplinary team and the contribution and global transcendence that this can imply, being able to improve the world around them. From both communication and scientific perspective, the international nature, the constant search for innovation in the project, as well as the social work committed to a health awareness challenge, such as AMR, make SWICEU a necessary project to bring science closer to society as a whole in a playful and educational way, also providing innovative solutions to this worldwide health issue that AMR represents [European Centre for Disease Prevention and Control (ECDC), 2020].

The SWICEU project is characterized by the creation of innovative pedagogical and didactic tools for disseminating the global health challenge posed by bacterial resistance to antibiotics among young people. This project has been internationally awarded, and financed with public and own funds used for dissemination and self-innovation purposes. It has been spread by the media and its content has reached high visibility on the Internet and social networks. This innovative approach can be summarized in the following five aspects:

1. The contents designed in English and Spanish by the SWICEU team have stood out internationally, being awarded on several occasions by the SWI and Tiny Earth networks, based in the United States, which bring together universities and educational centers participating in both projects in more than 15 countries (Tiny Earth, 2018, 2019).
2. The SWICEU team’s campaigns have achieved significant media and digital outreach with public health content designed to raise citizen awareness, reaching a potential audience of close to 5 million people (Actualidad CEU, 2020).
3. The development of gamification supports, web content, and content for social media have been designed to awaken scientific and informative vocations among young people about a global health challenge of maximum relevance (Actualidad CEU, 2021a).
4. The use of multiple digital platforms has also been combined with participation in real face-to-face scientific experiences: experiments in schools and scientific events at the University (Actualidad CEU, 2019, 2021b).
5. The variety of formats used, especially in the recreational and online media, have been designed to appeal especially to young audiences: card games, board games, and games that encourage creativity, online contests and challenges, YouTube videos, infographics, social media formats (stories, polls, contests, and threads), informative content with videos and image galleries, scientific events at the University, etc.

## Conclusion

The initiatives compiled in this manuscript have been the result of the effort of the SWICEU team during 5 years dedicated to improve concepts related to Health Sciences, and more specifically on infectious diseases, AMR, and the rational use of antibiotics. All the strategies have been created from the team’s own vocation, thus representing the relevance of spreading the importance of knowing these issues from different ways, making society understand that we can all contribute to improve the situation through adequate knowledge and awareness. With the same strategies we have approached to the population, and more specifically to future university students, both the university world and STEM careers, together with careers such as Journalism. All the results obtained reflect the importance of creating novel, creative strategies, and approaches adapted to the whole society in order to raise awareness and inform the population about such an important global health problem as AMR.

## Data availability statement

The original contributions presented in the study are included in the article/supplementary material, further inquiries can be directed to the corresponding author.

## Ethics statement

Written informed consent was obtained from the individual(s) for the publication of any potentially identifiable images or data included in this article.

## Author contributions

M-TP-G was responsible for the project design, conception, and management, the integrity of the work, and overall supervision, performed the practicals, and wrote sections. AT-P, BS-G, CG-R, and JB-B performed the practicals. EM-C was responsible for the broadcasting of the project. AT-P and EM-C wrote the first draft of the manuscript. BS-G, EM-C, CG-R, and JB-B wrote sections. All authors contributed to the article and approved the submitted version.

## Funding

This project was supported by grants CEU INNOVA Programme (PI14B-SV-17, PI09B-SV-18, 342 PI68B-SV-19-20, and PI53B-SV-21) and the Vice-Rectorate for Research (grants INDI18/34, INDI 19/39, INDI20/38, and INDI21/44) from Universidad CEU Cardenal Herrera and Fundación Española para la Ciencia y la Tecnología (FECYT), Ministerio de Ciencia e Innovación (FCT-19-14737), España. A-TP was supported by CEINDO-SANTANDER (Spain).

## Conflict of interest

The authors declare that the research was conducted in the absence of any commercial or financial relationships that could be construed as a potential conflict of interest.

## Publisher’s note

All claims expressed in this article are solely those of the authors and do not necessarily represent those of their affiliated organizations, or those of the publisher, the editors and the reviewers. Any product that may be evaluated in this article, or claim that may be made by its manufacturer, is not guaranteed or endorsed by the publisher.
